# Microscopic Droplet Size Analysis (MDSA) of “Five Thieves’ Oil” (Olejek Pięciu Złodziei) Essential Oil after the Nebulization Process

**DOI:** 10.3390/molecules28114368

**Published:** 2023-05-26

**Authors:** Wojciech Smułek, Maciej Jarzębski, Marek Ochowiak, Magdalena Matuszak, Jan Kaczorek, Jerzy Stangierski, Jarosław Pawlicz, Paweł Drobnik, Piotr T. Nowakowski, Joanna Dyrda-Muskus, Grzegorz Fiutak, Mieczysław Gorzelak, Sirsendu S. Ray, Kunal Pal

**Affiliations:** 1 Institute of Chemical Technology and Engineering, Poznan University of Technology, Berdychowo 4, 60-695 Poznań, Poland; 2 Poland Department of Physics and Biophysics, Faculty of Food Science and Nutrition, Poznań University of Life Sciences, Wojska Polskiego 38/42, 60-637 Poznań, Poland; 3 Independent Researcher, 60-965 Poznań, Poland; 4 Department of Food Quality and Safety Management, Faculty of Food Science and Nutrition, Poznań University of Life Sciences, Wojska Polskiego 31/33, 60-624 Poznań, Poland; 5 Department of Orthopedics and Traumatology, Poznan University of Medical Sciences, 28 Czerwca 1956 135/147, 61-545 Poznań, Poland; 6 Institute of Pedagogy, Rzeszów University, Jałowego 24, 35-010 Rzeszów, Poland; 7 Department of the Branch Office in Stalowa Wola, The Catholic University of Lublin, 37-450 Stalowa Wola, Poland; 8 Department of Biotechnology and General Technology of Food, Faculty of Food Technology, University of Agriculture in Krakow, Balicka 122, 30-149 Krakow, Poland; 9 Department of Orthopedy and Rehabilitation, Medical University of Lublin, 20-059 Lublin, Poland; b.leszczynska@umlub.pl; 10 Department of Biotechnology and Medical Engineering, National Institute of Technology, Rourkela 769008, India

**Keywords:** microscopy, droplet size, nebulization, essential oils, *P. aeruginosa*

## Abstract

Nowadays, due to a higher resistance to drugs, antibiotics, and antiviral medicaments, new ways of fighting pathogens are intensively studied. The alternatives for synthesized compositions are natural products, most of which have been known in natural medicine for a long time. One of the best-known and intensively investigated groups are essential oils (EOs) and their compositions. However, it is worth noting that the method of application can play a second crucial part in the effectiveness of the antimicrobial activity. EOs possess various natural compounds which exhibit antimicrobial activity. One of the compositions which is based on the five main ingredients of eucalyptus, cinnamon, clove, rosemary, and lemon is named “five thieves’ oil” (Polish name: olejek pięciu złodziei) (5TO) and is used in natural medicine. In this study, we focused on the droplet size distribution of 5TO during the nebulization process, evaluated by the microscopic droplet size analysis (MDSA) method. Furthermore, viscosity studies, as well as UV-Vis of the 5TO suspensions in medical solvents such as physiological salt and hyaluronic acid, were presented, along with measurements of refractive index, turbidity, pH, contact angle, and surface tension. Additional studies on the biological activity of 5TO solutions were made on the *P. aeruginosa* strain NFT3. This study opens a way for the possible use of 5TO solutions or emulsion systems for active antimicrobial applications, i.e., for surface spraying.

## 1. Introduction

Essential oils (EOs) can be obtained from almost all parts of plants, e.g., seeds, flowers, stems, bark, etc. EOs are secondary metabolites which are very important for the plant’s overall defence mechanism and also possess various medicinal properties [1]. About 10% of all known essential oils are commonly used due to their aroma and currently, 3000 types can be identified [2]. According to the OEC (The Observatory of Economic Complexity), in 2020, the market for essential oils was estimated at USD 5.41 billion, which places it in 454th place in the most in-demand products in the worldwide market [3]. The most commonly produced is orange oil, followed by corn, mint, and citrus oil [4].

Essential oils (EOs) are one of the most intensively studied natural compositions due to their antimicrobial activity. Recent studies showed that EOs possess rich sources of various chemical volatiles, such as ketones and aldehydes, e.g., cinnamaldehyde [5], terpenoids [6], anthocyanins [7], and more than 100 single other substances, including phenylpropanoids and short-chain aliphatic hydrocarbon derivatives [8]. For that reason, EOs are considered to be potentially antibacterial, antiviral, insecticidal, and larvicidal, as well as a preservation agent for food, cosmetics, and biomedical applications [9,10,11,12,13].

It must be highlighted that EOs and their components were intensively studied as antiviral agents for SARS-CoV-2. Strub et al. performed molecular docking studies of EOs and isolated natural compounds to be used for designing SARS-CoV-2 protease inhibitors [14]. Some studies suggested that a combination of EOs with chemical substances may be a more effective agent in the fight against the COVID-19 virus, especially due to diffusion into the lungs [15]. It should be noted that direct inhalation of EOs, i.e., during nebulization, is generally not recommended. However, new solutions, i.e., based on nanosized structures [16] delivered during nebulization for lung diseases, are needed. In this regard, medicinal plants are of particular interest [17].

Some of the EOs evaporate rapidly, which limits their applications, especially as antimicrobial agents. Due to this fact, carrier systems for EOs have been developed. Hu et al. [18] proposed microcapsule-based maltodextrin, whey protein isolate, and sodium alginate for cinnamon EO prepared with spray drying. For further controlled release, coacervation or solid lipid nanoparticles can be applied as well [19].

One of the most common techniques for EOs’ stability improvement is emulsion preparation. In recent years, microemulsions and nanoemulsions [20,21,22] have become more and more popular. For the decrease of emulsion droplet size, ultrasounds are frequently applied [23,24]. A review prepared by da Silva et al. [25] showed that the final antibacterial activity of the nanoemulsion systems can be a distinguished key factor, such as composition and droplet size, as well as power, sonication time, and ultrasound treatment procedure. It corresponds with other studies [26,27] where the effectiveness of active antimicrobial agents applied on the surface, as well as during therapies, such as aerosol therapy or inhalation, depending on the applied liquid droplet size, was presented.

In the previous study [28], the “five thieves’ oil” (Polish name: olejek pięciu złodziei) composition (5TO), which is based on eucalyptus, cinnamon, clove, rosemary, and lemon Eos, was characterized. In that study, spectroscopic tests (FTIR, UV-Vis, and fluorescence) and chromatographic analyses were performed, followed by contact angle and custom of L*a*b* colour measurements; this was then concluded with bacterial activity tests. Based on previous results, we decided to extend our research to the possible application of 5TO as a potential surface-active antimicrobial agent. At this stage, we focused mainly on microscopic droplet size analysis of the droplets produced during nebulization. The nebulizer was used as a device for the manufacturing of repeatable droplet size, which can be considered a possible surface-spraying technique. Moreover, for further studies, UV-Vis spectra of the 5TO dispersions were registered.

## 2. Results and Discussion

**Figure 1:**
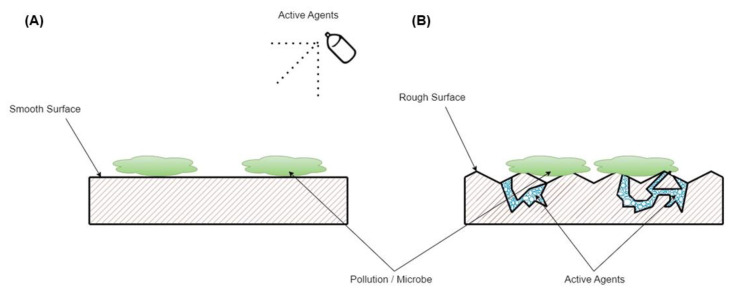
Schematic presentation of the possible use of EOs as a surface antimicrobial agent: (**A**) EOs sprayed at the surface and (**B**) long-term evaporation on a rough surface.

Mahanta et al. [29] prepared an overview of recent strategies in the development of antimicrobial surfaces. They divided current research on surfaces into four main topics: (i) smart surfaces, (ii) functionalized, (iii) superwetable, and (iv) patterned. In opposition to the previously used common smooth surface where a typical active agent is sprayed (see scheme in Figure 1), the rough surface with a controlled microchannel could be applied for the long-term release of antimicrobial liquids. It might be beneficial in healthcare units, where staff numbers are decreasing, and in unusual stations, such as a pandemic, for which their amounts of duties increase. As was mentioned before, as an alternative to synthetic compounds, EOs are of particular interest as an active antimicrobial agent. Figure 1b shows the idea of a rough surface with a microchannel for the controlled release of antimicrobial liquid. It should be noted that, typically, droplet size distribution (in emulsion systems, colloids, or dispersions) is studied using “artificial” conditions, such as in ultrapure water, with additional surfactant addition, or after filtration. Moreover, the most frequently used techniques which are applied for these studies are dynamic (rather static) light scattering, confocal laser scanning microscopy, or electron microscopy (the most frequently in cryo mode). Concerning non-standard conditions, such as the application of an active agent dispersed in a liquid (i.e., emulsion or dispersion system) onto the surface, the most important problem is how and what kind of apparatus can be applied. Here, we propose microscopic droplet size analysis (MDSA) for a droplet size evaluation of 5TO EOs. The presented results can be considered a milestone in the development of new methods for evaluating surface active agent examination.

### 2.1. Spectrometric Studies and Refractive Index

**Figure 2:**
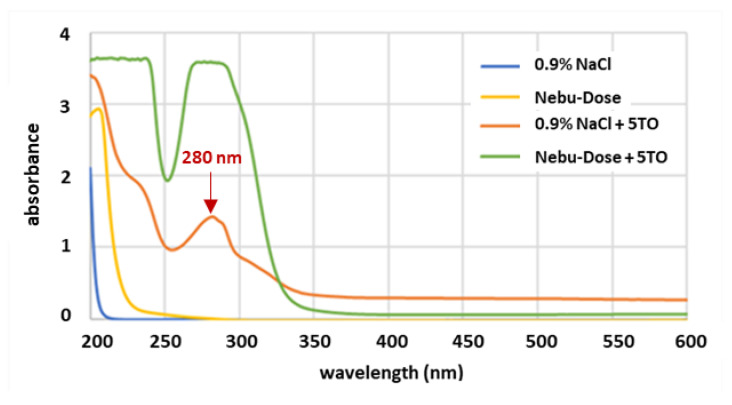
UV-Vis spectra of tested solutions and dispersions.

In this study, we dispersed 5TO in NDH and NaCl solutions. In opposition to the typical emulsion system [30], we did not add any surfactants or other stabilizers. It is for this reason that we named our systems dispersions. The first tests focused on the properties of dispersions intended for spraying. Figure 2 shows the UV-Vis spectra for 0.9% NaCl solution and NDH solutions with and without the addition of 0.5% 5TO. It can be observed that the signals from 0.9% NaCl and NDH are quite high, and the absorbance is over 2. We decided to study the samples as delivered, without additional dilution, to reflect natural conditions as much as possible. To reduce the signal from 5TO, in Figure 2, we present samples with concentrations of 0.5%.

The spectra of basic solutions contain no specific signals. This also indicates high light transmittance in the analyzed range of light wavelengths. Significant changes in absorbance are evident only when essential oils are introduced into the mixed systems. As stated by Kucharska-Ambrożej et al. [31], essential oils’ UV-Vis spectra have strong signals between 240 and 270 nm. This region typically corresponds to π→π* and n→π* electron transfer, and the n→π* transitions most likely arise from bonds in terpene molecules. In our study, the samples with 0.5% oil content possess distinct signals with a local signal maximum at 283 nm, which corresponds to the absorbance of terpene present in compounds in the oil mixture such as eugenol [32], which is one of the main components of the five thieves’ oil [28]. They include, among others, eugenol [32], cinnamaldehyde [33], and α-pinene [34]. In addition, the oils may contain some amounts of natural pigments, chlorophyll, and carotenoids, which show absorbance in the observed range of signals [35]. The higher signal intensity for the Nebu-Dose samples compared to the 0.9% NaCl sample indicates a greater dispersion of the oil phase in the water, which may be due to the stabilizing effect of hyaluronic acid. The signals for the samples containing 0.5% of the oil were too intense and exceeded the measuring range of the spectrophotometer. It can be assumed that, in addition, interactions were formed between the terpenoid compounds present in the oils comprising 5TO and hyaluronic acid. They may have the character of hydrogen bonds, the existence of which may indirectly affect the dissipation of the energy of transitions between the valence electron states mentioned above. Nevertheless, this is a working hypothesis that requires separate research.

**Figure 3:**
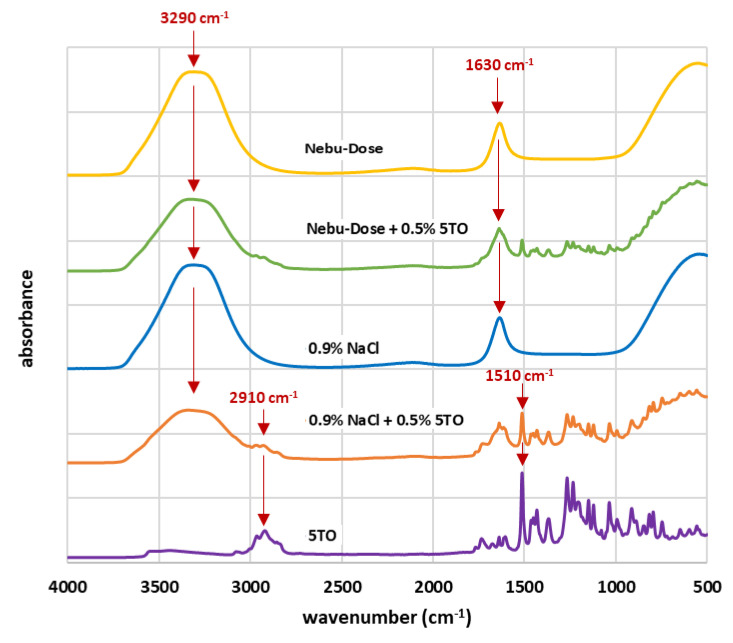
Infrared spectra of tested solutions and emulsions.

Detailed studies of the 5TO composition were presented in our previous study [28], where GC-MS (gas chromatography and mass spectra analysis), FTIR (Fourier-Transform Infrared Spectroscopy), UV-Vis, and fluorescence behavior were evaluated. The FTIR spectra of nebulization solutions are presented in Figure 3. The dominant signals at 3290 cm^−1^ and 1630 cm^−1^ can come from water molecules in the liquid phase. In the 5TO spectrum, signals comes from the C-H bonds’ vibrations (e.g., between 2800 cm^−1^ and 3000 cm^−1^) or carbonyl group (at ca. 1500 cm^−1^). What is interesting is that the spectra of Nebu-Dose show no signals from hyaluronic acid. The spectra from solutions containing 5TO are a direct composite of a set of signals from 5TO and 0.9% NaCl and Nebu-Dose base solutions.

Further studies focused on characterizing the optical properties of solutions and emulsions. Table 1 shows the values of the refractive index (RI) and turbidity. It can be observed that the presence of hyaluronic acid in Nebu-Dose increased the RI compared to samples with only NaCl. However, the effect of the addition of 5TO was nondenominational, as it resulted in a decrease in the RI for the isotonic NaCl solution and, in turn, an increase for the Nebu-Dose solution. By observing the turbidity values, it can be seen that they increase from a level not exceeding 1 (for solutions without 5TO) to nearly 150 for the 5TO emulsion in NaCl and nearly 200 for 5TO in Nebu-Dose. The higher turbidity value for the latter system can be explained by the stabilizing effect of hyaluronic acid. The turbidity of emulsions is directly correlated with the droplet size [36]. The particle size limit between a transparent and cloudy (turbid) emulsion is considered to be around 100–250 nm [37,38]. In addition, the acidity of the test samples was also examined (Table 1). Nebu-Dose solutions were more acidic than NaCl solutions. At the same time, when 0.5% 5TO was added, it contributed to a slight increase in pH.

### 2.2. Rheological Test Results

**Figure 4:**
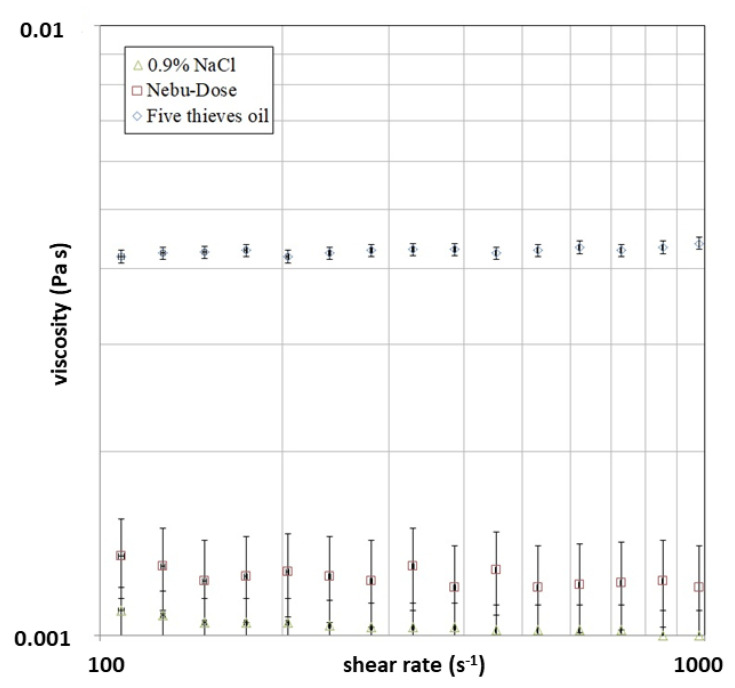
The viscosity of tested solutions vs. shear rate.

The performed overview of the literature shows that there are still not enough data on the EOs’ viscosity, as well as EOs’ compositions. The studies performed by Silva et al. [39] on EOs show that the viscosity of oils/resins from *Copaifera multijuga* ranges from 9.46 to 92.62 mPaꞏs. In Figure 4, the relation between the viscosity of tested solutions and the shear rate is presented. The performed analysis showed that all of the dispersion behaved like a typical Newtonian liquid. The registered dynamic viscosity for NDH was 0.0012 ± 0.0002 Pa·s; for 0.9% NaCl, it was 0.0010 ± 0.0001 Pa·s.; and for 5TO, it was 0.0042 ± 0.0001 Pa·s. Due to fast evaporation, it is difficult to perform viscosity measurements for most of the EOs, and the data presented here can be applied, i.e., for further spray or nebulizer systems’ development. It should be highlighted that the viscosity affects the droplet formation (this is discussed in the following sections) and that the droplet size is one of the key factors for the antimicrobial properties of the applied surface liquids. Furthermore, viscosity measurements performed in environmental conditions are supportive for designing new antimicrobial agents; i.e., these could be applied to prevent hospital or health service infections. Noteworthy is the fact that EOs are considered an alternative to commonly used antibacterial and antiviral agents for antibiotic-resistant bacteria, such as *Klebsiella pneumoniae* [40].

As earlier stated, additional carrying systems for EOs, such as emulsions, impact their evaporation, as well as rheological parameters.

### 2.3. Microscopic Droplet Size Analysis (MDSA)

Typically, the droplet size distribution of emulsions, suspensions, or dispersions is evaluated by dynamic light scattering (DLS), which allows some systems to detect the signals in the range of 0.1–10,000 nm [41,42]. Hence, it should be noted that the DLS is a “blind” technique, which registers the scattered light signals from particles or droplets in the analyzed dispersion without distinguishing what the analyzed structure is. Probably, air bubbles or other contaminations can be detected as a particle or droplets. Furthermore, previous studies of the emulsions systems showed that microscopic studies of the emulsions give information about the additional processes taking place in the sample, such as coalescence [43], which can be “interpreted” by DLS as a larger particle occurring and, thus, impact the final particle/droplet size determination [44]. On the other hand, microscopic imaging is limited due to resolution, so a combination of various analysis techniques is recommended for a detailed characterization of the dispersion. The benefit of the MDSA technique is its application in the droplet size distribution on different surfaces.

**Figure 5:**
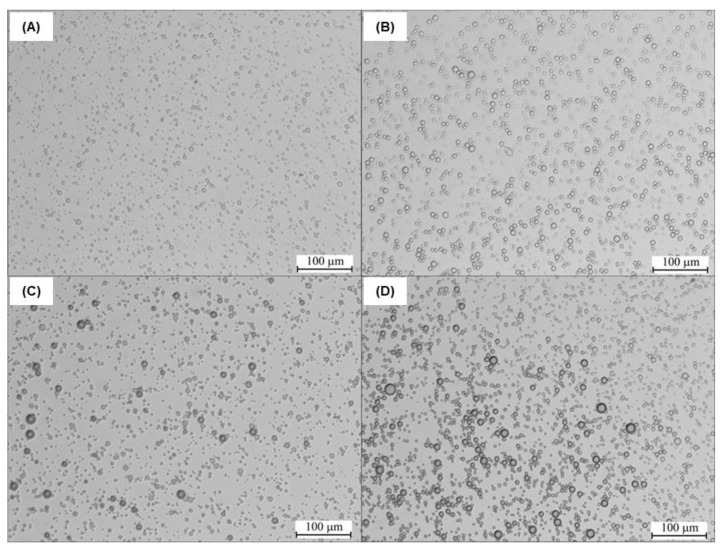
Sample microscopic images of sprayed solutions: (**A**) 0.9 % NaCl, (**B**) 0.9 % NaCl + 0.5% 5TO, (**C**) NDH, and (**D**) NDH + 0.5% 5TO.

Figure 5 shows examples of microscopic images obtained during MDSA of aerosol droplets obtained as a result of spraying the tested solutions. It has been observed that the addition of 5TO contributes to obtaining an aerosol with larger droplets. The spraying process is complex and depends on many parameters (macroparameters and microparameters), which are closely related to each other. The macroparameters are related to the characteristics of the external shape and internal structure of the droplet stream, while the microparameters concern the qualities of the spray [45]. The mean droplet diameters are conventional values included in the group of microparameters. The average diameter of the droplets is one of the most important values that enable a comparative assessment of the spray quality. It is worth emphasizing that this parameter may define various values, which include the diameter, area, number of or volume of drops. There are many types of mean droplet diameters, the value of which is determined by the main formula of the form [45]:

(1) $$D_{ab}={\left[\frac{\sum_{i=1}^{m}N_{i}\cdot D_{i}^{a}}{\sum_{i=1}^{m}N_{i}\cdot D_{i}^{b}}\right]}^{1/(a-b)}$$

where the exponents *a* and *b* represent a specific diameter. In the literature, the size of the generated aerosol droplets is usually determined by the volume–surface diameter of the droplet D_32_ (SMD, Sauter Mean Diameter) and the median D_0.5_ (MMD, Mass Median Diameter; or CMD, Count Median Diameter) [46]. The average diameter of D_0.5_ defines exactly 50% of the mass, volume, or number distribution of the droplets [45]. The mean diameter D_32_ defines the diameter of a homogeneous substitute set containing the same total volume and the area of all droplets corresponding to the real set. D_32_ enables the description of many important processes, including mass and heat transfer [46].

The second parameter often described in the literature is the particle size distribution. There are surface, number, volume, and mass distributions [46]. A particle size distribution is a static set of droplets where the random variable is the droplet diameter (D). It is assumed that the spectrum of the distribution of droplet diameters defines the relationship between the number of Ni droplets in the interval of

(2) $$D\in \langle D_{i}-\frac{\Delta D}{2},\;D_{i}+\frac{\Delta D}{2}\rangle$$

where *Di* is the diameter that corresponds to the middle of each *i*-th interval, and Δ*D* is the constant width of the interval [46].

Table 2 shows a summary of selected analyzed mean droplet diameters for the tested solutions. It was noticed that, for the NDH solution and 0.9% NaCl solution with the addition of 0.5% of 5TO, the analyzed mean droplet diameters for the spraying process carried out in a pneumatic nebulizer were greater than the diameters obtained for pure solutions. Moreover, it was observed that, for all the tested solutions, the mean droplet diameters were ≤5 μm. Considering the statistical significance of the differences between the results of the series of measurements for individual samples, it can be concluded that, for some of the parameters studied, i.e., D_30_, D_10_, and D_0.5_, the effect of the addition of 5TO, rather than the presence of hyaluronic acid, was significant. What is interesting is that, for the parameters D_32_ and D_43_, no statistically significant differences can be seen between the samples with and without 5TO and in the samples with NDH. The overview of the collected results indicates that the determination of all molecular diameters of *D* gives a better picture of the average particle size. However, in the studied case, it can be concluded that the introduction of 5TO to a sprayed mixture lead to an increase in average droplet size.

**Figure 6:**
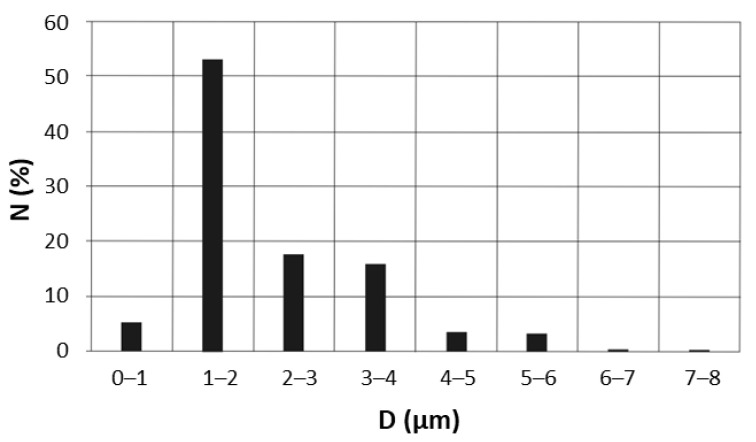
Droplet size distribution of 0.9% NaCl solution after nebulization, determined by microscopic studies.

**Figure 7:**
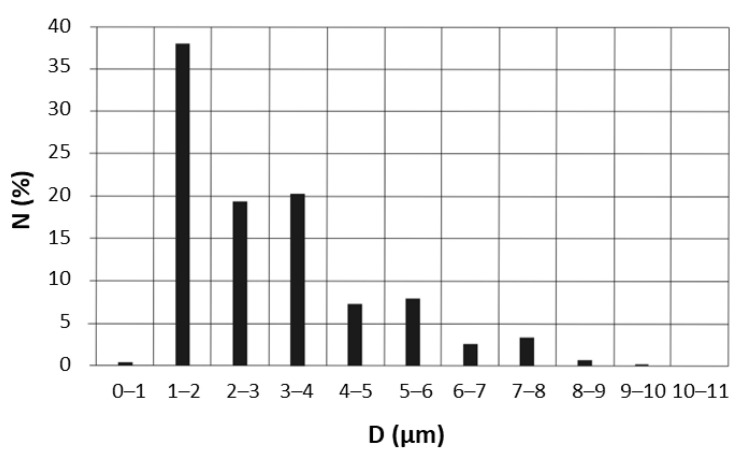
Droplet size distribution of 0.9% NaCl with 5TO solution after nebulization, as determined by microscopic studies.

**Figure 8:**
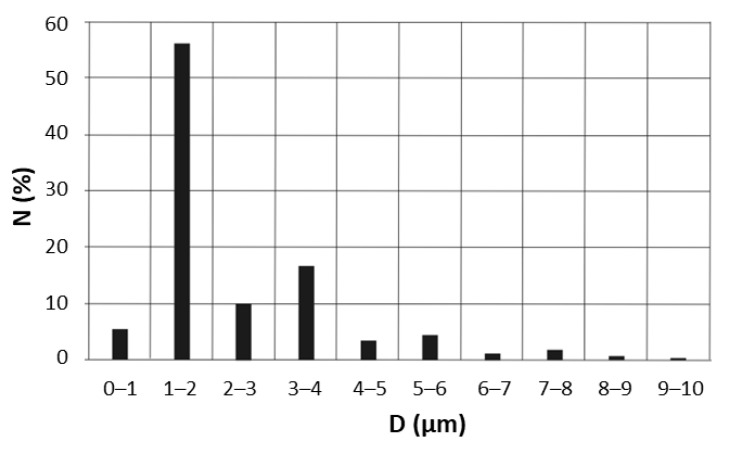
Droplet size distribution of NDH solution after nebulization, as determined by microscopic studies.

It should be noted that the EOs’ emulsions and nanoemulsions are frequently prepared using a two-step procedure, where in the first step, the composition is vigorously stirred with surfactant or stabilizer, using a homogeniser, and then ultrasound treated [47]. The ultrasound treatment decreases the droplet size even into the nano-range [48] and improves the emulsion stability. In this study, we mixed 5TO with NaCl solution and NDH, using only a homogeniser, without any surfactants. That high impact on the size of the droplets is presented in Figure 6, Figure 7 and Figure 8. The numerical distribution obtained with MDSA showed that the main droplet fraction of the generated aerosol was observed in the range of 1–2 μm droplets. Furthermore, the obtained numerical distribution of drops proves that the addition of 5TO to the base solutions causes an increase in the number of larger drops and a reduction in the number of the smallest drops and leads to their expansion. This proves that additional tests of droplet size distribution after spraying are necessary for the further design of antimicrobial systems—for the final droplet size may affect the presence of salt—here from NaCl solutions.

### 2.4. Surface Properties

**Figure 9:**
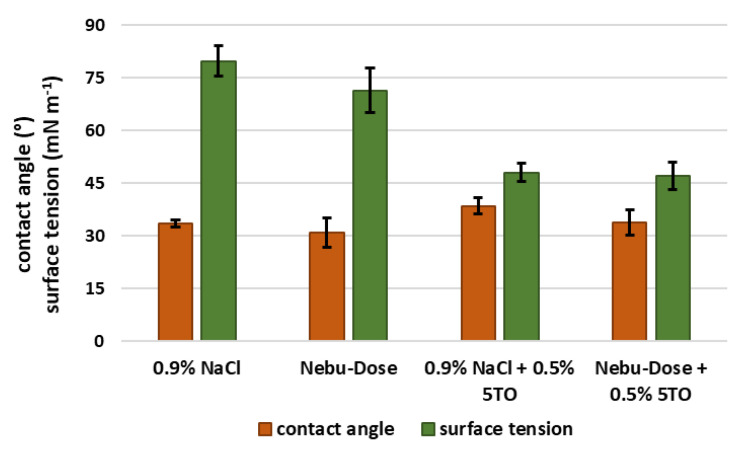
Results of contact angle and surface tension measurements of the dispersions.

**Figure 10:**
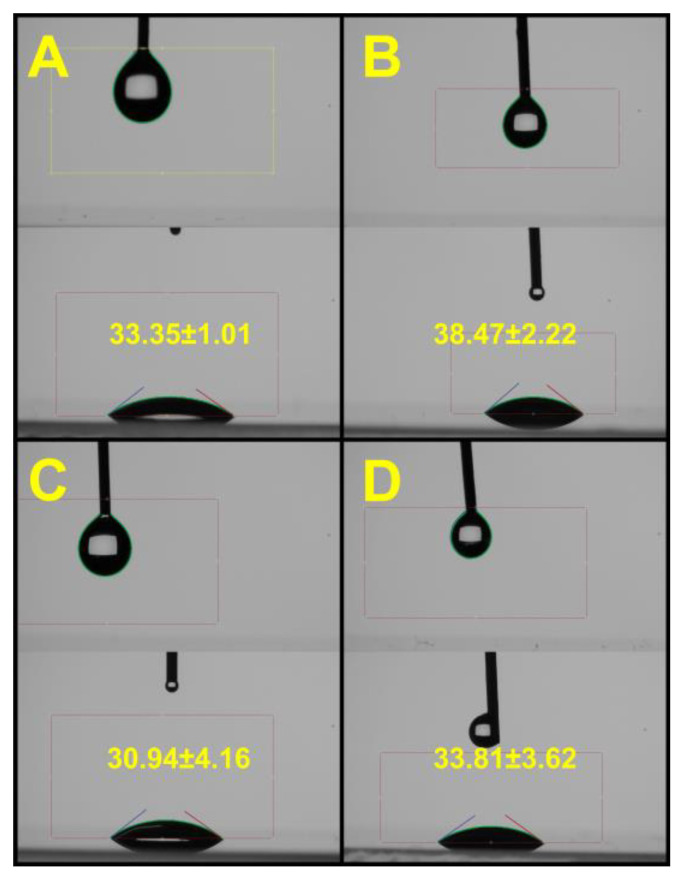
Pictures taken during contact angle (downside) and surface tension measurements (pending droplet measurements—upper side), (**A**)—0.9% NaCl, (**B**)—0.9% NaCl + 0.5% 5TO, (**C**)—Nebu-Dose, (**D**)—Nebu-Dose + 0.5% 5TO.

The next stage of the study was the analysis of the surface properties of the droplets of the base solutions and the 5TO emulsions obtained on their basis. The obtained results are shown in Figure 9 and Figure 10. The values of the wetting angle on the glass oscillated from 31° (for NDH) to 38° (0.9% NaCl + 0.5% 5TO). It can be concluded that the presence of hyaluronic acid caused a slight decrease in the wetting angle, while the addition of 5TO minimally increased it. Much more pronounced were the differences between the post-surface tension of the measured samples. The values of this parameter for the systems without 5TO were similar to the surface tension of pure water solutions, with hyaluronic acid causing a marked reduction in the value from 79 mN/m to 71 mN/m. They very strongly affected the surface tension by the presence of 5TO in the samples. The measured parameter then took values of about 47 mN/m.

### 2.5. Biological Activity

Our preliminary tests of 5TO showed that the compositions of eucalyptus, cinnamon, clove, rosemary, and lemon EOs affect cell activity and showed antibacterial activity [28]. It must be pointed out that 5TO’s composition effectiveness was different than the separately used essential oils from rosemary, lemon, clove, eucalyptus, and cinnamon.

**Figure 11:**
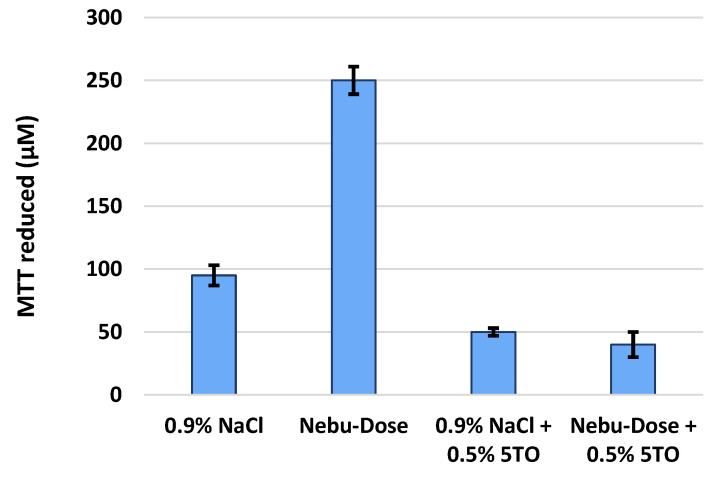
Metabolic activity of *Pseudomonas aeruginosa* NFT3 strain in tested solutions.

Further research was devoted to evaluating the antibacterial properties of the mixtures used in the spray tests. The obtained results of the cell metabolic activity test of *P. aeruginosa* strain NFT3 are shown in Figure 11. The experiment proved that investigated mixtures with essential oils exhibit strong antibacterial properties. In the method used, the higher the concentration of reduced MTT, the higher the metabolic activity of the cells. The bacteria cell activity in 0.9% NaCl solution was on the level of 95 μM of MTT reduced and decreased over 40% when 5TO was added. For NDH samples, the decrease in microbial activity caused by the addiction to 5TO was even more visible. As observed, the cell activity in samples with 5TO was nearly 85% lower than the cell activity in the sample with NDH only. What should be pointed out is that the relatively high activity of the bacteria in samples with NDH can be explained as a result of the use of hyaluronic acid as a substrate by bacteria. The results correspond with the research of Denkova-Kostova et al. [49] or Alexa et al. [50], who also confirmed the strong activity of clove and lemon oils, respectively, against *P. aeruginosa*. This allows us to conclude that the obtained aerosols containing the oil of the five thieves have significant bactericidal properties and may be used in the future in aerosol therapy of the upper respiratory tract.

It is noteworthy that the bactericidal effect of EOs is observed only above a certain defined level, below which they show a rather bacteriostatic effect, or they do not affect the growth of pathogenic microorganisms at all, as found by Leja et al. [12] in their study with EOs extracted from juniper fruits (*Juniperus communis* L.), lemongrass leaves (*Cymbopogon citratus*), rosemary leaves (*Rosmarinus officinalis*), and black pepper (*Piper nigrum*). The microorganisms they tested were the bacterial strain Pseudomonas orientalis P49 and Pseudomonas orientalis P110. Moreover, when comparing 5TO with the EOs reported in the literature, it is important to remember that it is a mixture of five EOs from different sources. Each of the constituent EOs may exhibit different effects on bacterial cells of bacterial strains, as found by Siejak et al. [28], using four bacterial strains for testing: *Pseudomonas* sp. MChB, *Pseudomonas aeruginosa* Pa2, *Pseudomonas plecoglossicida* IsA, and *Pseudomonas* sp. 02.1. In the described work, the authors found that the most intense antibacterial activity was presented by clove oil (leading to over 50% decrease in cell metabolic activity), cinnamon oil, and 5TO. However, the other EOs present in 5TO had, when used separately, a non-toxic effect and even, to some extent, stimulated the metabolic activity of the bacterial cells.

## 3. Materials and Methods

### 3.1. Materials

The oil composition (5TO), which is traditionally called five thieves’ oil (in Polish, “olejek pięciu złodziei”), was purchased from Natura Receptura (Elbląg, Poland). As a solvent for 5TO Nebudose, 0.9% NaCl (which will be called NaCl solution) and Nebudose 0.1% hyaluronic acid water solution (which will be called NDH) were purchased from Solinea (Poland). For the tests, all reagents were used as delivered, without further purification.

### 3.2. Oil Dispersion Preparation

A total of 0.5% of 5TO was suspended in 0.9% NaCl solution, as well as NDH. An IKA homogenizer with an S18N-19G/03.502546 tip was used to produce the dispersion. The homogenization process was carried out at a constant speed of 6000 RPM for 10 min.

### 3.3. Methods

#### 3.3.1. UV-Vis and FTIR

The UV-Vis spectra of the basic solutions (0.9% NaCl and NDH) and their dispersions with 5TO were prepared using a V-750 spectrophotometer (Jasco, Tokyo, Japan) within the wavelength range 200–800 nm. Moreover, the infrared spectra in the range of 4000–500 cm^−1^ were obtained using a Spectrum Two FTIR spectrometer equipped with a Universal ATR with a diamond crystal (PerkinElmer, Waltham, MA, USA).

#### 3.3.2. Rheological Tests

The dynamic viscosity of the liquid compositions was tested using an Anton Paar Physica MCR 501 rheometer (Anton Paar, Graz, Austria). The device was equipped with air bearings for the measurement of torque in the range from 0.01 µNm to 300 mNm and the rotation frequency from 10^−5^ to 628 rad/s. The device was operated in a measuring system of coaxial cylinders. The dual gap DG26.7/SN21100 measurement systems (Anton Paar, Graz, Austria) were used. The temperature was stabilized by employing the Peltier system at a temperature of 20 °C, with an accuracy of 0.01 °C. The program was set for the shear in the range of 105–1020 1/s for 500 s.

#### 3.3.3. Nebulization/Droplets Spraying

A nebulizer with a spray head of MedelJet Basic (Medel Group S.p.a., San Polo di Torrile, Italy) was used to carry out the research. This setup was equipped with a pneumatic nebulizer (compressor nebulizer), and a container with a nominal volume of up to 6 mL.

#### 3.3.4. Microscopic Droplet Size Analysis (MDSA)

A microscopic droplet size analysis (MDSA) was performed using a biological research Nikon Eclipse 50i microscope (Nikon, Tokyo, Japan). The microscope was set on a stable surface that was not exposed to sunlight, which could cause disturbances in the image. Before use, the device was subjected to appropriate framing (scaling) to select the appropriate scale. A 10× lens was used. The images were collected using an Opta-Tech camera (Opta-Tech, Warsaw, Poland) (with a resolution of 2048 × 1536 pixels). The obtained images were processed in the program Image-Pro Plus (MediaCybernetics Inc., Rockville, MD, USA), using the diameters of the formed droplets that were measured.

The MDSA analysis of the nebulization process includes two elements:

(1) Visualization of the sprayed liquid structure;
(2) Statistical evaluation of the size of the generated droplets.

The MDSA technique presented here is based on the method of catching drops on the immersion liquid, which, in this case, was a layer of oil (oil 20–90, produced by the Institute of Petroleum Technology in Kraków, Poland). The oil layer was evenly distributed on a glass plate to investigate the diameters of the aerosol. Then, the sprayed aerosol (of 5TO dispersions) during nebulization was captured on a 76 × 26 mm (thickness of 0.95 mm) flat glass microscope plate.

Here, nebulization was carried out with a nasal tip. The distance between the nose tip and the glass plate with the immersion liquid was the same for each measurement series and was about 2 cm. A simple wire with the possibility of adjusting its position and length was attached to the tip of the nose with a clamp, and a plate was placed on its other end, at an appropriate distance.

#### 3.3.5. Surface Properties

To evaluate the surface properties of the nebulization solutions, an Ossila contact angle goniometer (Ossila Ltd., Sheffield, UK) was applied. One droplet of the selected solutions was dropped onto the microscopic glass surface for the contact angle measurements. For surface tension measurements, a glass syringe with a 25 µL volume and a blunt-tipped needle of 0.47 mm in diameter was applied. The average values of 5 measurements were presented. For the pendant droplet measurements, the droplet density was uniformly adjusted by software for water The measurements were performed at room temperature.

#### 3.3.6. Refractive Index and Turbidity

A PAL-RI optical electronic refractometer (ATANGO CO., LTD, Tokyo, Japan) was used for refractive index (RI) determination. The RI of the dispersions was collected at room temperature. Averages and SD values of 5 repetitions of the measurements were presented.

#### 3.3.7. pH Measurements

The pH measurements were performed using a Seven2Go^TM^ pH meter equipped with an InLab Solids Go-ISM electrode (Mettler-Toledo). The pH meter was calibrated before the measurements, using Rainbow I pH 4.01/7.00/9.21 standards (Mettler-Toledo, Warsaw, Poland).

#### 3.3.8. Biological Activity Tests

To evaluate the bactericidal activity of the nebulized liquids’ microbial activity, a test was conducted with *Pseudomonas aeruginosa* strain NFT3 (NCBI GenBank No.: MK493328). Cells were incubated in 24 h culture in nutrient broth at 30 °C and then washed and re-suspended in the broth to obtain optical density at 600 nm ca. 1.0. Then, 25 µL of the solution was mixed with 20 µL 5 mg L^−1^ MTT solution and with 175 µL of Nebudose or 0.9% NaCl with or without 0.5% of five thieves’ oil. After 2 h of incubation at 30 °C, the samples were centrifuged, the supernatant was removed, and then the precipitate was dissolved in isopropanol and centrifuged again. Then, the supernatant absorbance at 590 nm was measured. The MTT (3-4,5-Dimethylthiazol-2-yl)-2,5-Diphenyltetrazolium Bromide) assay is based on a transformation of yellow MTT into its violet derivative, which is catalyzed with respiration enzymes in cells. The higher the transformation ratio the higher the viability of the cells.

### 3.4. Statistical Analysis

In all analyses, at least three independent experiments were performed, unless otherwise indicated. The mean values with standard deviation (SD) were accepted as the final results. Analysis of Variance (*p* < 0.05) was applied to compare variances across means (or average) of different data groups.

## 4. Conclusions

The studies of droplet size after nebulization that were performed with the microscopic droplet size analysis (MDSA) techniques presented in this paper can be considered support for the development of a new antimicrobial active agent. It should be highlighted that MDSA allows us to study the samples’ behaviour on the surface in “real” conditions, as this is beneficial for the evaluation of the interactions, i.e., surface active agent and further speed of evaporation. The performed viscosity tests of EOs’ dispersions might be applied for the design and development of new spraying systems. What is more, the results of the surface activity of the dispersions showed that even a low amount of EOs decrease their surface tension. Moreover, the pH values were decreased after the addition of the 5TO composition. The acidic environment is beneficial in the fight against bacterial infections. The presented results open a new perspective for the continuation of the research regarding the possible use of EOs as potential antimicrobial agents.

## Figures and Tables

**Table 1:** Properties of tested solutions and dispersions—refractive index, turbidity, and pH. Different small letters indicate samples statistically different, at *p* < 0.05.

Sample Description	Turbidity (-)	Refractive Index (-)	pH (-)
0.9 % NaCl	0.95 ± 0.17 (a)	1.3345 ± 0.0002 (a)	5.35 ± 0.30 (a)
0.9 % NaCl + 0.5% 5TO	145.20 ± 9.18 (b)	1.3338 ± 0.0001 (b)	5.60 ± 0.30 (a)
NDH	0.45 ± 0.05 (c)	1.3347 ± 0.0002 (a)	4.56 ± 0.35 (b)
NDH + 0.5% 5TO	201.80 ± 2.68 (d)	1.3352 ± 0.0001 (c)	4.90 ± 0.40 (b)

**Table 2:** Average droplet size after nebulization with SD; different small letters indicate samples statistically different, at *p* < 0.05.

Sample Description	*D*_10_ (µm)	*D*_32_ = *SMD* (µm)	*D*_30_ = *VMD* (µm)	*D*_43_ (µm)	*D*_0,5_ = *CMD* (µm)
0.9 % NaCl	2.30 ± 0.21 (a)	3.60 ± 0.29 (a)	2.90 ± 0.26 (a)	4.26 ± 0.34 (a)	1.93 ± 0.19 (a)
0.9 % NaCl + 0.5% 5TO	3.14 ± 0.30 (b)	5.67 ± 0.51 (b)	3.92 ± 0.39 (b)	4.85 ± 0.44 (a)	2.57 ± 0.26 (b)
NDH	2.40 ± 0.24 (a)	4.53 ± 0.32 (c)	3.33 ± 0.23 (a)	5.54 ± 0.33 (b)	1.93 ± 0.17 (a)
NDH + 0.5% 5TO	3.04 ± 0.21 (b)	4.62 ± 0.37 (c)	3.75 ± 0.38 (b)	5.54 ± 0.39 (b)	2.57 ± 0.21 (b)

## Data Availability

All that were collected during the test were presented here.

## Sample Availability

All samples which were investigated here are available on the market.
